# The soybean-Phytophthora resistance locus *Rps1*-k encompasses coiled coil-nucleotide binding-leucine rich repeat-like genes and repetitive sequences

**DOI:** 10.1186/1471-2229-8-29

**Published:** 2008-03-19

**Authors:** Hongyu Gao, Madan K Bhattacharyya

**Affiliations:** 1 Department of Agronomy, Interdepartmental Genetics, Iowa State University, Ames, Iowa 50011, USA

## Abstract

**Background:**

A series of *Rps *(resistance to *Pytophthora sojae*) genes have been protecting soybean from the root and stem rot disease caused by the Oomycete pathogen, *Phytophthora sojae*. Five *Rps *genes were mapped to the *Rps1 *locus located near the 28 cM map position on molecular linkage group N of the composite genetic soybean map. Among these five genes, *Rps1*-k was introgressed from the cultivar, Kingwa. *Rps1*-k has been providing stable and broad-spectrum *Phytophthora *resistance in the major soybean-producing regions of the United States. *Rps1*-k has been mapped and isolated. More than one functional *Rps1*-k gene was identified from the *Rps1*-k locus. The clustering feature at the *Rps1*-k locus might have facilitated the expansion of *Rps1*-k gene numbers and the generation of new recognition specificities. The *Rps1*-k region was sequenced to understand the possible evolutionary steps that shaped the generation of *Phytophthora *resistance genes in soybean.

**Results:**

Here the analyses of sequences of three overlapping BAC clones containing the 184,111 bp *Rps1*-k region are reported. A shotgun sequencing strategy was applied in sequencing the BAC contig. Sequence analysis predicted a few full-length genes including two *Rps1*-k genes, *Rps1*-k-1 and *Rps1*-k-2. Previously reported *Rps1*-k-3 from this genomic region [1] was evolved through intramolecular recombination between *Rps1*-k-1 and *Rps1*-k-2 in *Escherichia coli*. The majority of the predicted genes are truncated and therefore most likely they are nonfunctional. A member of a highly abundant retroelement, *SIRE1*, was identified from the *Rps1*-k region. The *Rps1*-k region is primarily composed of repetitive sequences. Sixteen simple repeat and 63 tandem repeat sequences were identified from the locus.

**Conclusion:**

These data indicate that the *Rps1 *locus is located in a gene-poor region. The abundance of repetitive sequences in the *Rps1*-k region suggested that the location of this locus is in or near a heterochromatic region. Poor recombination frequencies combined with presence of two functional *Rps *genes at this locus has been providing stable *Phytophthora *resistance in soybean.

## Background

Many plant disease resistance (*R*) genes from different plant species have been isolated and characterized; but are grouped into a limited number of classes [2,3]. *R *loci are usually organized in clusters, and genes within one cluster are mostly derived from a common ancestor [4]. The clustering feature can facilitate the expansion of *R *gene number and the generation of new *R *gene specificities through recombination and positive selection [5]. Long contiguous sequences containing several *R *genes or resistance gene analogues (RGA) have been determined [6-8]. These sequences provided insights into the mechanisms of *R *gene evolution and generation of novel recognition specificity. Insertions of retroelements in genomic regions containing *R *genes or RGAs have been documented in these studies. Retroelements are suggested to create variability among paralogous *R *gene members [9].

Soybean (*Glycine max *L. Merr.) is a legume crop of great economic and agricultural importance across the world. Its estimated genome size is 1,115 Mb, of which approximately 40–60% is composed of repetitive sequence [10-12]. Repetitive DNA sequences have been shown to be the major determinant of plant genome sizes [13]. There are two main types of repetitive sequences, tandem repeat DNA sequences and dispersed DNA sequences such as retroelements [13]. Several tandem repeats, SB92, STR120 and STRR102 have been reported in soybean [14-16]. It has been suggested that soybean has experienced at least two rounds of genome-wide duplications [17-19]. Despite the availability of genomics resources such as densely saturated genetic maps, BAC and YAC libraries, large EST collections, BAC end sequences, a soybean genome database (SoyGD) browser, and the legume information system (LIS) [20], our knowledge of soybean genome structure is still largely limited [21-27].

Root and stem rot disease caused by *Pytophthora sojae *is one of the most destructive soybean diseases in the United States [28]. Use of *Phytophthora *resistance conferred by single dominant *Rps *genes has been providing reasonable protection of soybean against this pathogen. Five *Rps *genes including *Rps1*-k were mapped to the *Rps1 *locus located near the 28 cM map position on molecular linkage group N of the composite genetic soybean map [29,30]. Among these five genes, *Rps1*-k was introgressed from the cultivar, Kingwa. *Rps1*-k confers resistance to most races of *P. sojae*, and has been widely used for the past two decades [31]. By applying a positional cloning approach two classes of functional coiled coil-nucleotide binding-leucine rich repeat (CC-NB-LRR)-type resistance genes were isolated from the soybean *Rps1-*k locus [1]. A large cluster of highly polymorphic paralogous *Rps1*-k sequences is located at the adjacent *Rps1*-k region [32]. The *Rps1*-k locus was mapped to two overlapping BAC clones encompassing 184 kb, located at one end of an approximately 600 kb contiguous DNA spanned by several overlapping BAC clones [32]. CC-NB-LRR-type genes of the 184 kb *Rps1*-k region were evaluated and two classes of highly similar genes were shown to confer race-specific Phytophthora resistance [1]. To gain insights into the soybean genome organization and evolution of *Rsp1*-k genes, BAC clones encompassing the *Rps1 *locus were sequenced and analyzed.

## Results

### Sequence of three BAC clones spanning the *Rps1*-k locus

*Rps1*-k was previously mapped to a region flanked by two markers CG1 and 18R [30,32]. To understand the composition of the *Rps1*-k region, three overlapping BAC clones, GS_18J19, GS_43D16 and GS_99I16 that may encompass the *Rps1 *locus were chosen for sequencing [32]. Phytophthora resistance genes were previously identified from these three BAC clones through positional cloning [1].

A total of 4,093 reads (829, 1,189 and 2,065 reads for GS_18J19, GS_43D16 and GS_99I16, respectively) were generated from these BAC clones. GS_18J19, GS_43D16 and GS_99I16 were sequenced to a 14-, 12- and 9-fold redundancies, respectively. A single contig of 38,498 bp was obtained for GS_18J19 after the initial assembly (GenBank accession EU450800). Three and five contigs were obtained from assembling of sequences derived from GS_43D16 and GS_99I16, respectively. The resulting contigs of GS_43D16 and GS_99I16 were ordered into individual scaffolds manually, in which the order and orientation of the contigs were inferred by mate pairs (sequences obtained from both ends of a ~20 kb shotgun clone) [33]. The clones that span the gaps between two adjacent contigs were identified based on mate pairs and were used to obtain sequences of the gap regions. Gaps were filled out by applying the primer walking approach. Primers were designed based on the sequences of contig ends from which walking were initiated. To guarantee the high sequence quality, less sequenced regions were further sequenced by using suitable primers. After initial assembly and gap filling, 70,829 and 164,411 bp sequences were obtained from GS_43D16 and GS_99I16, respectively (GenBank accession EU450800). The assembled GS_18J19 sequences represent one end of the GS_43D16.

### Directional sequencing of GS_43D16

Earlier, partial sequencing of the three BAC clones had allowed us to identify candidate genes underlying *Rps1*-k. The functional identities of *Rps1*-k genes were confirmed through stable transformation in soybean [1]. Two classes of *Rps1*-k genes were identified. The three Class I *Rps1*-k genes were identical in their ORF sequences. The Class I gene, *Rps1*-k-3, showed a recombination breakpoint at the 3' untranslated region originating from sequence exchange between members of both classes of genes [1].

**Figure 1:**
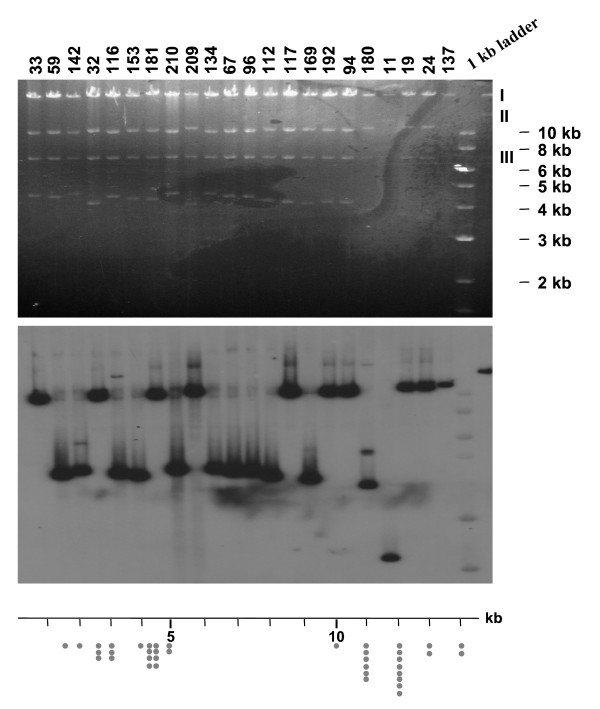
**Physical mapping of the locations of individual EZ::TN <*Not*I/KAN-3> transposon insertions in a soybean bacterial artificial chromosome**. Individual GS_43D16 clones containing the EZ::TN <*Not*I/KAN-3> transposon were digested with *Not*I. Three *Not*I fragments, I, II and III released from *Not*I digestion of GS_43D16 are shown in the last lane. Note that fragment III is comprised of the pBeloBAC11 vector sequence. The top panel shows the gel of *Not*I digested DNA of GS_43D16 clones carrying the transposon in the *Not*I Fragment II. The middle panel shows the Southern hybridization data of the gel shown in the top panel. The 245 bp probe for Southern analysis was obtained by PCR of the GS_43D16 end that overlaps with GS_18J19, but not GS_99I16. The lower panel shows the distribution of clones carrying the transposon at various regions of the *Not*I Fragment II. One dot represented one clone containing the transposon at that particular location of the *Not*I Fragment II.

*Rps1*-k-1, *Rps1*-k-2 and *Rps1*-k-3 were isolated from GS_43D16 [1]. The existence of abundant repetitive sequences made it difficult to assemble the sequences of the BAC clones. To avoid any misassembly, GS_43D16 containing *Rps1*-k-1, *Rps1*-k-2 and *Rps1*-k-3 was subjected to directional sequencing using the EZ::TN <*Not*I/KAN-3> transposon of the EZ::TN in-Frame Linker Insertion Kit (Epicentre, Madison, WI). Two hundred and twenty-four EZ::TN <*Not*I/KAN-3> transposon insertion GS_43D16 clones were randomly selected for further analysis. This approach allowed us to map physically the individual sequence reads onto the GS_43D16 sequence as follows. Transposon insertion sites of individual transposition events were utilized to select EZ::TN <*Not*I/KAN-3> transposon containing GS_43D16 clones. Each clone was digested with *Not*I and hybridized to GS_43D16 end-specific probes in Southern analyses (Figure 1). There are three *Not*I sites in GS_43D16; one in the insert soybean genomic DNA and two in the pBeloBAC11 vector flanking the cloning *Hin*dIII site. Therefore, *Not*I digestion of GS_43D16 resulted in three *Not*I fragments (Figure 1); (I) a large DNA fragment of ~55 kb, (II) a small DNA fragment of ~15 kb, and (III) the pBeloBAC11 vector sequence. There are two *Not*I sites flanking the kanamycin resistance gene in the EZ::TN <*Not*I/KAN-3> transposon. Therefore, if there is a single transposon insertion in the GS_43D16 clone, then five fragments including the ~1.2 kb transposon, should be generated following *Not*I digestion (Figure 1).

Of the analyzed 224 random transposon-inserted GS_43D16 clones, 162 were shown to contain the transposon in the large fragment; 40 of them in the small fragment; and 22 in the pBeloBAC11 vector. Clones containing transposon insertions in the vector pBeloBAC11 were not considered for further study. Approximate physical locations of transposon insertions in individual *Not*I genomic DNA fragments were determined by Southern analyses as shown in Figure 1. Based on the physical location of transposon insertions, 114 GS_43D16 clones containing transposon insertions in either the 15 or 55 kb *Not*I fragment were selected for sequencing by using transposon end-specific primers. Only about 50% percent of the clones produced sequences that were readable. Pairwise sequence comparison between the assembled GS_43D16 sequence and sequences obtained from individual transposon inserted GS_43D16 clones revealed the transposon insertion sites in GS_43D16.

Among the randomly picked 224 transposon-inserted clones, the number of transposon insertions was proportional to the size of *Not*I fragments. However, there were no insertions in two regions, one of about 5 kb in the ~15 kb fragment and the other one is about 10 kb in the ~55 kb fragment. Whether this was due to bias in transposon insertion or due to sampling variance is yet to be determined.

**Figure 2:**
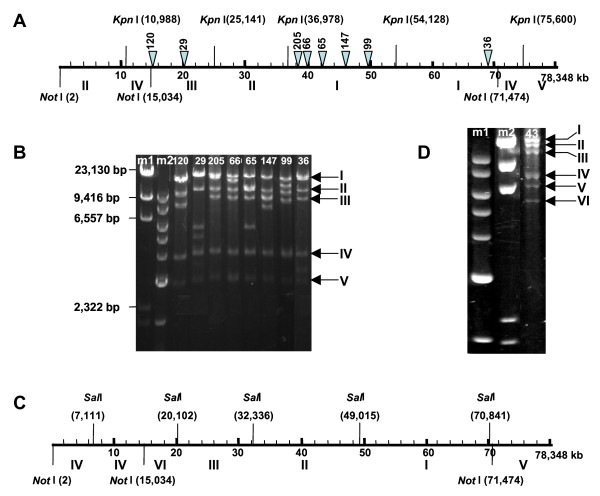
**Verification of the restriction maps of GS_43D16**. **A**, *Kpn*I and *Not*I map of the assembled GS_43D16 sequence. **B**, *Kpn*I and *Not*I double digestion of selected GS_43D16 clones carrying the EZ::TN <*Not*I/KAN-3> transposon insertions. Eight fragments were expected from *Kpn*I and *Not*I digestion of GS_43D16 carrying no transposons (**A**). Only five fragments were observed, because some of the fragments had similar mobilities in the gel. Some of these fragments were resolved after transposon insertion. A close relationship was observed between the restriction fragment sizes determined by gel electrophoresis and that by sequence data and location of transposon insertions (Table 1). m1, λ*Hin*dIII ladders, m2, 1 kb DNA ladder (New England Biolabs Inc., Beverly, MA). **C**, *Sal*I-*Not*I map of the assembled GS_43D16 sequence. **D**, *Sal*I and *Not*I digestion of GS_43D16. Eight fragments were expected from the double digestion of GS_43D16 (Figure 2C). Six fragments were resolved from the digestion of the clone (43 in 2D). 7.9 kb and 7.11 kb fragments were not resolved (Fragment IV, twice the intensity of either Fragment III or Fragment V) and 0.6 kb *Sal*I-*No*tI fragment is not included in 2D.

The quality of 78,313 bp assembled GS_43D16 sequence was verified through restriction mapping as follows. Clones carrying transposon insertions at various regions were selected and double digested with *Kpn*I and *Not*I. The predicted *Kpn*I – *Not*I restriction maps based on the assembled GS_43D16 sequence is shown in Figure 2A. Eight fragments are expected from *Kpn*1 and *Not*I double digestion of GS_43D16. Only five fragments were resolved in the gel analyses, because some of the fragments are of similar sizes. For example, there are two 17 kb fragments termed Fragment I. Following digestion of clones carrying single transposons with both enzymes released two additional fragments and the 1.2 kb transposon. Depending upon the position of the transposon in a given *Kpn*I or *KpnI*-*Not*I fragment two fragments of variable sizes were produced (Table 1). Comparison of observed fragment sizes with that of expected fragment sizes showed that there is general agreement between the observed and expected fragment sizes. *Sal*I-*Not*I map (Figure 2C) based on the assembled sequence was also verified by digesting GS_43D16 with *Sal*I and *Not*I. Eight fragments were expected from the double digestion. Two fragments, 7.9 kb and 7.11 kb were not resolved and termed Fragment IV (Figure 2C and 2D). Smallest fragment (0.6 kb) is not shown in Fig 2D. Taking these data together, it was concluded that the generated GS_43D16 sequence represents the physical distance of the soybean DNA present in that clone and no large fragments were remained to be sequenced.

**Table 1:** Restriction fragments produced from the *Kpn*I-*Not*I double digestion of GS_43D16 clones carrying the EZ::TN <*Not*I/KAN-3> transposon.

		**Fragment size from *Kpn *I/*Not*I double digestion^2^**	
		
**Clone ID**	**Location of the transposon^1^**	**Observed**	**Expected^3^**
120	15,171	~10 kb, -^4^	0.1 kb, 10.1 kb
29	20,255	~5.5 kb, ~4.5 kb	5.2 kb, 4.9 kb
205	38,493	~15 kb, -	15.6 kb, 1.5 kb
66	39,577	~14.6 kb, 2.6 kb with Fragment 3.	14.6 kb, 2.6 kb
65	42,203	~11.9 kb, 6 kb	11.9 kb, 5.2 kb
147	45,914	~10 kb with Fragment 3, ~8 kb	8.9 kb, 8.2 kb
99	49,522	~12.5 kb, 4.6 kb with Fragment 7.	12.5 kb, 4.6 kb
36	68,935	~14.5 kb, ~3 kb	14.8 kb, 2.5 kb

^1^The sequence flanking the transposon of the transposon inserted GS_43D16 clones were compared with the assembled GS_43D16 sequence.

^2 ^Fragments originating from transposon insertion.

^3^The expected fragment sizes based on the restriction map of the assembled GS_43D16 sequence and location of transposon insertion.

^4^The small fragment was not detected.

### Genes underlying the *Rps1-k*

**Figure 3:**
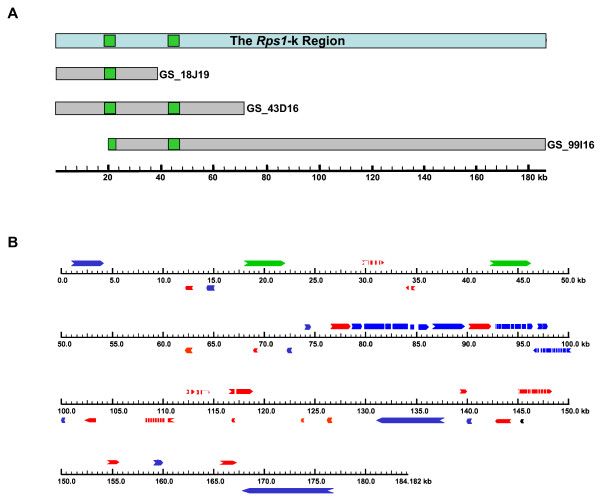
**Molecular characterization of the *Rps1*-k region**. A) The overlapping three BAC clones containing two CC-NB-LRR genes (green box) of the *Rps1*-k region are depicted. Sequences of individual BACs were utilized to show their overlapping regions. The composite *Rps1*-k region, shown at the top of the figure, is based on the sequences of these three BACs. GS_99I16 does not carry the 5'-end of *Rps1*-k-1, which is shown with a truncated green box. B) Arrangement of predicted genes and retrotransposons in the Rps1-k region. The green colored boxes represent full-length genes (*Rps1*-k-1 and *Rps1-*k-2); the red colored boxes represent partial genes; the blue colored boxes represent retroelements; white boxes represent introns in the predicted genes. Boxes above the ruler represent genes that have coding sequence on the forward strand, whereas the boxes under the ruler indicate the genes that are on the reverse strand. Detailed annotation data are presented in Table 2.

GS_18J19 overlaps with one end of GS_43D16. GS_99I16 comprised 51,109 bp sequences of GS_43D16 (Figure 3). There were 99.99%, 99.85% and 99.96% identities between the overlapping sequences of GS_18J19 and GS_43D16, GS_43D16 and GS_99I16, and GS_18J19 and GS_99I16, respectively. These results indicate high quality of the assembled sequences. High identity of GS_43D16 sequence with the overlapping regions of GS_18J19 and GS_99I16 suggested that there was no rearrangement in GS_43D16, from which *Rps1*-k-1, *Rps1*-k-2 and *Rps1*-k-3 were previously isolated [1].

The gene content of an 184,111 bp contig sequence (GenBank Accession Number EU450800) carrying the *Rps1*-k locus derived from the GS_43D16 and GS_99I16 sequences was determined. Genes were predicted with GeneScan and GeneMark.hmm ES-3.0 programs [34]. To get a better gene prediction, genes predicted by GeneScan and GeneMark.hmm, and/or sequences having similarities to soybean ESTs were further analyzed by different NCBI Blast programs and sequence alignment programs. Putative annotations of the predicted genes were accomplished by BlastP searches. The gene content in the *Rsp1*-k region appears to be poor. Only a few full-length genes were predicted. These include two coiled coil-nucleotide binding-leucine rich repeat (CC-NB-LRR)-type *Rps*1-k genes and retrotransposons (Figure 3, Table 2).

**Table 2:** Gene annotations of the *Rps1*-k region^1^

**Gene ID**	**Position^2^**	**Predicted gene annotation**	**Closest protein homolog**	**BLASTP** **E value**	**Soybean ESTs^3 ^(E ≤ e^-50^)**
1	18019–21708 (+)	*Rps1*-k-1	Glycine max *AY963292*	0	14
2	42452–46201 (+)	*Rps1*-k-2	Glycine max *AY963293*	0	14
3	63302–62705 (-)	CBL-Interacting protein kinase 15	Arabidopsis thaliana *NP_195801*	6e^-69^	4
		Serine/threonine Kinase (partial seudogene)	Persea Americana AAL23677	3e^-68^	
4	69126–68921 (-)	Ribosomal protein S6	Glycine max *AAS47511*	4e^-7^	17
5	76950–78815 (+)	Conserved hypothetical protein	Medicago truncatula *ABD32262*	3e^-40^	4
6	79282–86280 (+)	Gag/pol polyprotein	Pisum sativum *AAQ82033*	0	19
7	90317–92266 (+)	Hypothetical 65 kDa avirulence protein in avrBs3 region	Xanthomonas campestris *pv*. vesicatoria *P14729*	5e^-5^	7
8	92658–96559 (+)	Gag-pol polyprotein	Zea Mays *AAM94350*	2e^-147^	21
9	113419–113916 (+)	NADH dehydrogenase subunit 1 (only the N-terminal 70 aa)	Trichosurus vulpecula *NP_149931*	3.9	77
10	114088–115118 (+)	MAD2 (only the N-terminal 65 aa)	Triticum aestivum *BAD90977*	3e^-17^	5
11	117048–116804 (-)	Cytochrome c oxidase subunit II (the N-terminal 40 aa)	Cynomys ludovicianus *AAK52712*	5.1	2
12	117709–119789 (+)	Cysteine proteinase Vacuolar processing enzyme precursor (the N-terminal 118 aa)	Glycine max BAA06030 P49045	3e^-49^	13
13	123937–123409 (-)	Unknown protein (partial pseudogene)	Arabidopsis thaliana *NP_190603*	2e^-26^	3
14	127141–126821 (-)	L-lactate dehydrogenase (partial pseudogene)	*Lycopersicon esculentum CAA71611*	9e^-27^	7
15	131753–138850 (-)	Glycoside hydrolase Integrase, catalytic region (partial pseudogene)	Medicago truncatula *ABD33337 *Medicago truncatula ABD32527	0	6
16	139054–139575 (+)	Unnamed protein product (C-terminal 173 aa)	Oryza sativa *NP_912905*	5e^-60^	3
17	140361–140014 (-)	Gag-pol polyprotein	Glycine max *AAQ73529*	1e^-34^	3
18	145152–148184 (+)	Dynein	Oncorhynchus mykiss *CAA33503*	1e^-10^	4
19	145722–145277 (-)	Prion-like Q/N-rich domain protein PQN-33	Gallus gallus *XP_428546*	6e^-48^	3
20	154355–155745 (+)	Oxidoreductase (pseudogene)	Arabidopsis thaliana *NP_201530*	2e^-21^	7
21	159487–160392 (+)	Gag/pol polyprotein	Pisum sativum *AAQ82037*	5e^-28^	9
22	165713–166447 (+)	Glycoside hydrolase, family 1, Zinc finger, CCHC-type; Ribonuclease H fold	Medicago truncatula *ABD333337*	6e^-29^	1
23	167488–176781 (-)	SIRE1–8 retroelement	Glycine max *AY205610*	0	5

^1^Genes not having matched soybean ESTs were not included.

^2^ indicates the coding sequence is on the forward sequence, while – indicates the coding sequence is on the reverse sequence.

^3^Soybean expressed sequence tags showing similarities to the target sequence at a level of significance, E value ≤ e^-50^

Most of the identified genes are truncated. Genes were considered truncated when their predicted reading frames are partial. For example, the predicted cysteine proteinase shares an 88% identity with the first 126 amino acids of a soybean cysteine proteinase protein (BAA06030) followed by a premature stop codon. BlastN search against the soybean EST database was performed to support our gene prediction. ESTs showing high similarities but no complete identities to all predicted genes were identified (Table 2).

### The *Rps1*-k region is composed of repetitive sequences

The major portion of the contiguous 184,111 bp sequence of the *Rps1*-k region is comprised of repetitive sequences including simple repeat sequences, tandem repeats and retroelements. The simple repeat and tandem repeat sequences were identified using Sputnik and tandem repeats finder. Sixteen simple repeat sequences were identified (Table 3). Sixty-three tandem repeats were revealed with copy numbers ranging from 1.8 to 72 and unit length varying from 7 to 310 bp (Table 4). The consensus motif length of the tandem repeat containing 72 copies is 24 bp. Sequence data from individual reads confirmed that they are tandem repeats in head-to-tail orientation. This sequence was used to query the soybean GSS (genomic survey sequence) database and a number of sequences with high identities were revealed. The one (CL868124) showing highest identity to the consensus 24 bp tandem sequence came from the project on characterization of the heterochromatic, gene-poor centric regions of soybean chromosomes.

**Table 3:** Simple repeat sequences in the *Rps1*-k region

**Position**	**Repeat Unit**	**Copy Number**
7619–7663	AT	22
9814–9851	AT	19
24196–24231	AT	18
34682–34732	AT	25
38898–38960	AAT	21
41328–41354	AAT	9
51716–51901	AT	93
53915–53944	AT	15
59145–59168	TC	12
64934–64989	AT	28
110292–110313	AT	22
112406–112477	AT	36
116097–116116	AT	10
116665–116714	AT	25
127258–127281	AG	12
181688–181759	AT	36

**Table 4:** Tandem repeat sequences in the *Rps1*-k region

**Position**	**Consensus sequence of tandem repeat unit**	**Copy number**
4872–4907	TTAATAAATTTATT	2.6
5279–5311	TTTATT	2.5
7219–7253	TTTTATTATTTAAATAT	2
7328–7366	TTTTAAGTTAACATAAATT	2
13986–14041	CTTATATTTTTTTTAT	3.5
14069–14121	TTTAAATCTTTTATTTTTACC	2.5
28228–28272	TTTATTTATAAGATTATTTAAT	2
34767–34826	ATGCAAACATATATACATGC	2.9
65181–65235	TCATTACTAAAAAAAAATAG	2.8
65966–66017	GCCAGCATGCATGTATATC	2.7
70677–70718	TAAAAAGTTGAATAGATAC	2.2
72634–72694	CATTAAGTTCTTTTAATTCCTAGGTTAGTGG	2
75090–75128	CGTTCTTCAT	3.8
87791–87926	TGAATATATATAGCATGAAAATGCCTTGCAAAATA	3.9
89787–89849	AAATAGAAAAGGAAAGAAAATG	2.9
90350–90511	AAAAAGAAAAGAAAGGAAATTCCCAATCAAAGAGAAAGC	3.8
90381–90538	GAGAAAGCAAAAAGAAAAGAAAGGAAATTCCCAATCAAAGAGTGG	3.5
91333–92076	TACGCGGAGATACCTTACGGTTATCCGCACCCCCTTTGCCATTCAGACACAGTCGTGTCCGTTGGCAAGCAGAGACCAAGTTTGGTCATTCTGCACACATGA	7.3
92743–92779	GCTCGCCTGGGCGAGCTGA	1.9
98273–98333	CATTAAGTTCTTTCAATTCATAGGTTAGTGG	2
113516–113540	AAAAACCGTCTTA	1.9
120828–120857	TTTTTTTTTCC	2.7
122442–122512	ATCAAATAAAATGCTTGCAGATCA	3
124367–124513	AAAAAAAATTGAAGATTCTAAGACAGTTTTTAGGGAAAACCGTCTTAGAATGTCTTATTTTAAATAAAAAAAAATT	2
133966–134004	AATCAAAGAACAACTCAAGTG	1.9
134057–134089	TCAAGAA	4.9
135918–136076	GATCCACAAGGGATGTACCCTCCCTTATTCTCATTACAACAACCCAAGTAGATGTACCCTCCACT	2.3
136235–136365	AAGGGAGAAGAGAGACACAAAAAGAATTCAGGCGGTTAGTCCTTGTCGATTCTTTTTGGAA	2.2
137034–137101	TCTTCTCTTGAATCTTGAATTCAA	2.9
144892–144919	AGAAAAGGAAAAA	2
145379–147112	GGACTACACGTCCTCGCCTTCAGA	72
147479–147972	GGGATCGCGCCCACAAGACACCCAGTGGACCCGAAGGAGTCCAACAGGGCCCTGGGGTTTCCAGCTCTGGTTACGGGCCTCTGTCAGTCCTACAGGGTGCCCGTCCCCCCCAGCAAGGTCACCCCATCGTAACATAGGTAACTATGCACATCTCTCAACTGATTTCTGATGCCATCCAATATTTGCA	2.6
148467–148657	AAAAATACCTCACAAAATATATATATATTATGTTTAGGTAGCAAGATACCTTGGATACACATGTATATAGC	2.7
149273–149361	AAAGAAAGTTCCCGATCAAAGATCGAAAGAAAACAAAGAGAAAA	2
150401–150651	GTATGGTTATCAGCACCTGTCGTCAACCAGGGGCAAACGAGCCCGTTGACGCGCAGAGACTAACGTCATCTTCTGCACCTTTTGTCAACCAGAGACAGCGAGTCCAATGACATGTGGAGATACCCAAGCGATTATCC	1.8
150612–151127	GCACCTTTTGTCATCCAGAGACAGCGAGTCCGATGACATGCGAGGGTACCGTATGGTTATCC	8.3
150799–150931	CACCTTTCGTCAACCAGGGGCAAACGAGCCCATTGACGCGCAGAGACTAACGTCGTCTTCTG	2.1
150550–151365	GCACCTTTCGTCAACCAGGGGCAAGCGAGCCCGTTGACGCGCAGAGACTAACGTCGTCTTCTGCACCTTTTGTCAACCAGAGATAGCGAGTCCGATGACATGCGAGGGTAACGTATGGTTATCCGCACCTTTTTTCATCCAGAGACAGCGAGTCCGATGACATGCGGGGGTACCGTATGGTTATCCGCACCTTTTGTCATCCACAGACGGCAAGTCCGATGACACGCGGAGGTACCGTATGGTTATCCACACCTTTCGTCAACCAGGGGCAAACGAGCCCATTGACGCACAGAGACTAACGTCGTCTTCC	2.6
150536–151060	CCGTATGGTTATCACACCTTTCGTCAACCAGGGGCAAACGAGCCCATTGACGCGCAGAGACTAACGTCGTCTTCTGCACCTTTCGTCAACCAGAGAGAGCGAGCCCAATGAATGCGAGGCTAACGATCGTTATCCGCACCTTTTATCATCCAGAGACGGCTAGTCCGATGACATGCGGGGGTACCGTATGGTTATCCGCACCTTTTGTCATCCACAGACAGCAAGTCCGATAACACGCAGGGGTA	2.1
150901–151386	CGCAGAGACTAACGTCGTCTTCCGCACCTTTTGTCATCCAGAGATAGCGAGTCCGATGACATGCGGAGGTACCGTATGGTTATCCGCACCTTTTGTCAACCAGAGGCAAGCGAGTCCGTTGACA	3.9
151985–152116	AATCCGTAAAGTTTCGCAACATTCTGGAAGTCAAAACAAGTATTGCTGCAC	2.6
152550–152596	TTCTTCATCG	4.6
152558–152597	CGTTCTTCATCGTTCTTCGTT	1.9
153216–153398	CCAAGAGATCGTTAATGGTCCAACGCCTTAACGTTTCTCTCCTTTCAAAA	3.6
153555–153596	AAAAAAGACAAAAAACAT	2.3
156483–156603	ATCAAACATCACTTGAGATCGTTTCAAGGTCCAACGCCTTAACCATTCTCTCCGCTTTTC	2
161707–161856	ACATCTGAGAAGAAAACTCATTCGACCAGGAGCTCATGGAAAATTCCCAAAGACAATTGTGATAGTAGGGT	2.1
162626–162804	TTTTAGAGGACTCAAAGTCCTCACCTTTATC	5.8
163722–163780	ATCAAAGAACAACTCAAGTGA	2.9
163762–163815	GAATCAAGAACAAGTCAAGACTCAA	2.1
163765–163818	TCAAGAATCAAGAAGAAT	2.9
163826–163911	AATCAAG	12.3
164281–164330	TTCAAAAAGGTTTTAACTTT	2.5
164461–164485	TTGAATCTCT	2.5
166799–166839	AGTATTTTCAAAAAT	2.9
168846–168891	TCATAAATCATGCATAATATCCT	2
172682–172713	TTTTCTGCA	3.4
178161–178219	ATCAAAGAACAACTCAAGTGA	2.9
178201–178254	GAATCAAGAACAAGTCAAGACTCAA	2.1
178204–178257	TCAAGAATCAAGAAGAAT	2.9
178265–178343	AATCAAG	11.3
178713–178762	TTCAAAAAGGTTTTAACTTT	2.5
180693–180809	AAAGGCACGCTAAGCCCAATTCCAACCGAGAGGAAGTGCACTGAGCGGCCC	2.3
183350–183378	AATTTATGGAGCCA	2.1

Another abundant tandem repeat contains the consensus AATCAAG sequence. There are 12.3 copies of this repeat sequence between positions 163,795 and 163,880 and 11.3 copies between 178,234 and 178,312. Several soybean tandem repeat sequences, SB92, STR120 and STR102, have been identified [14-16]. Seven copies of a tandem repeat sequence with 102 bp unit length were also found in the *Rps1*-k locus, but it shares no similarity with STR120 or STR102.

The ~20 kb intergenic sequence between *Rps1*-k-1 and *Rps1*-k-2 is primarily made up of repetitive sequences. Four simple repeat sequences were localized in this interval. Notably, a 220 bp sequence was found at two locations, one between positions 24,318 and 24,537 and the other one between positions 29,963 and 30,182. This sequence encodes part of a protein sharing high similarity to the receptor-like protein kinase, Xa21 (BAD27933).

A *copia*/*Ty1*-like retroelement, *SIRE1–8*, was identified from the assembled 184,111 bp sequence of the *Rps1*-k region [35]. The 9.5 kb sequence encoding the *SIRE1–8 *element was used to query the soybean EST database. Two ESTs (CB063565 and CO983516) showed 99% identity to part of the gag-pol encoding sequence, one EST showed 92% identity to the LTRs and one EST exhibited similarities to the envelope-like sequence.

### The CC-NB-LRR-type gene, *Rps1*-k-3 evolved through intramolecular recombination in *Escherchia coli*

Previously it was reported that a CC-NB-LRR gene, *Rps1*-k-3 was evolved from recombination between *Rps1*-k-1 and *Rps1*-k-2 [1]. The gene was comprised of 5' end from *Rps1*-k-1 and 3'-untranslated region from *Rps1*-k-2. This gene was isolated form GS_43D16 but not from either GS_18J19 or GS_99I16. This observation suggested that these two BAC clones did not overlap [1]. Therefore, it was concluded that the two Class I CC-NB-LRR genes isolated from GS_18J19 and GS_99I16 were unique.

**Figure 4:**
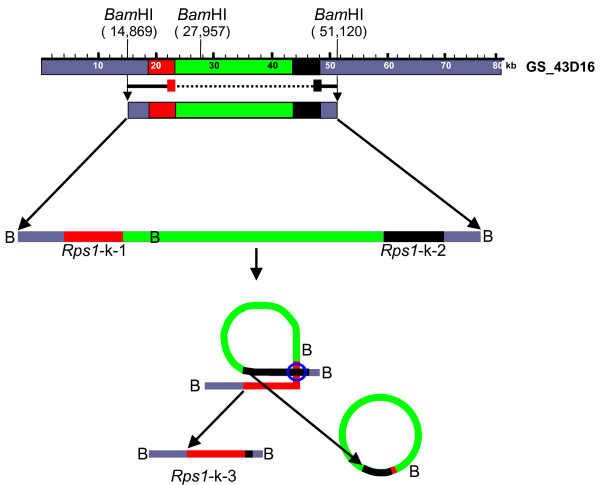
**Generation of the *Rps1*-k-3 through intramolecular recombination**. Locations of *Rps1*-k-1 and *Rps1*-k-2 on the GS_43D16 sequence are shown. Partial *Bam*HI digested GS_43D16 DNA was cloned into the binary vector pTF101.1 and a library of binary clones was obtained. The library was screened for LRR sequences. Binary clone p43-10 contained the *Rps1*-k-3 gene, which is not present in GS_43D16 (Figure 3). This gene was presumably originated from intramolecular recombination in *E. coli*. Three *Bam*HI (B) sites involved in generation of the *Rps1*-k-3 [1] are shown on the map. Solid line shows the region cloned in p43-10 and broken line indicates the region not found in p43-10. Presumably this region was lost during the recombination process in *E.coli*. The possible recombination process involved in the evolution of *Rps1*-k-3 is shown at lower part of the figure. The two identical 174 bp sequences of *Rps1*-k-1 (red line) and *Rps1*-k-2 (black line) involved in the recombination process are shown within the blue open circle (21,980 through 22,154 of *Rps1-*k-1 and 46,473 through 46,647 of *Rps1*-k-2 in the *Rps1*-k region shown in Figure 3B). The proposed model for the recombination event in *E. coli *is based on the article by Weisberg and Adhya [65].

Following sequencing, *Rps1*-k-3 was not identified from GS_43D16. Physical mapping of GS_43D16 (Figure 2) and high identities between overlapping sequences of three independent BAC clones (Figure 3) suggested strongly that the complete sequence of GS_43D16 was obtained and this clone did not go through any rearrangement. *Rps1*-k-3 was identified from a binary clone, p43-10 cloned in the pTF101.1 vector [1]. The clone was isolated from a library of binary clones prepared from GS_43D16 DNA partially digested with *Bam*HI. Since this gene was not found in GS_43D16, the gene must have evolved through intramolecular recombination in *Escherichia coli*. Comparison of the insert sequence of p43-10 with GS_43D16 sequence revealed that the insert of the binary clone carries sequences identical to a *Bam*HI fragment that contains an internal *Bam*HI site (Figure 4). However, p43-10 insert DNA does not contain a segment of the GS_43D16 including the internal *Bam*HI site. This gene presumably originated from recombination in *E. coli*. By looking at the recombinant breakpoint in *Rps1*-k-3, it was hypothesized that two identical 174 bp sequences (21,980 through 22,154 and 46,473 through 46,647 of the *Rps1*-k region shown in Figure 3B) of the 3'-end of both *Rps1*-k-1 and *Rps1*-k-2 were involved in the RecA-independent recombination process as shown in Figure 4.

The major recombination pathway in *E. coli *requires RecA [36]. Therefore, *recA- *strains, such as DH10B used in our experiment, are considered for recombinant gene cloning experiments to avoid any recombination events. Unfortunately, RecA-independent intraplasmid recombination does occur in these *recA- *strains. The frequency of recombination is however much lower as compared to that observed in *recA*+ strains [37,38]. A recent study suggested that RecA-independent recombination is suppressed by single-strand DNA exonuclease (ssExos) activity. In absence ssExos, the extent of RecA-independent recombination in *recA- *strains is comparable to that in *recA+ *strains [39].

## Discussion

Genomes of higher plants vary significantly in their size and complexity. Repetitive DNA sequences have been shown to be the major determinant of genome sizes in higher plants [13]. The prevalence of transposable elements and retroelements can promote unequal crossing-over leading to transposon-mediated rearrangements and gene duplications [40]. It has been hypothesized that transposable elements play a major role in the expansion and diversification of transmembrane receptor kinase-type disease resistance *Xa21 *gene family [9]. The abundance of retroelements has been observed in several genomic regions containing *R *genes or *RGA *loci, such as barley powdery mildew resistance gene, *Mla*, and *Citrus virus *resistance gene, *Ctv *[7,8]. The variability among 14 rice *Xa21 *gene members has been considered to be generated mainly from the rearrangements mediated by transposon-like elements [9]. *Rps1*-k genes are arranged closely. About 38 copies of *Rps1*-k-like sequences were predicted to exist in the soybean genome. Most of the copies are clustered in the *Rps1*-k region [32]. A *copia*-like retroelement, Tgm*r*, has previously been reported from the *Rps1*-k region [41]. It is possible that retrotransposons facilitated the amplification of the *Rps1*-k gene family.

In many plant species such as *Arabidopsis thaliana *and *Medicago tuncatula*, chromosome arms are differentiated into euchromatic and heterochromatic regions [42-44]. Recently, Lin et al. [14] showed that in soybean heterochromatic regions are also delimited from euchromatin. Studies in *Arabidopsis*, *Medicago *and *Lycopersicon esculentum *have shown that the euchromatin has a high gene density, whereas pericentromeric heterochromatin is largely comprised of repetitive sequences [44-46]. The *Rps1*-k region is composed of mostly tandem repeat sequences and retroelements (Figure 3; Table 2). The gene content is very similar to that of a soybean BAC clone identified from the pericentromeric heterochromatin [14]. FISH mapping showed that *SIRE1 *and other retroelements are sequestered to the heterochromatic and/or pericentromeric regions [14]. The tandem repeat sequences and retroelements including *SIRE1 *are commonly abundant in heterochromatic and/or pericentromeric regions of the soybean genome. Therefore, the *Rps1*-k region could be located in heterochromatic region which may be pericentromeric.

The possible microcollinearities of the *Rps1*-k locus with genomic sequences of plant species such as *Arabidopsis*, *Medicago *and *Lotus japonicus *were investigated. An R protein-like sequence of *Medicago *genomic clone, MTH2-138E10, showed 65% identities to Rps1-k-2. However, no synteny was observed between the *Rps1*-k region and MTH2-138E10 sequence. A limited synteny of the *Rps1*-k locus was observed with the *Lotus *genome. Two copies of a *Lotus Rps1*-k homolog located five kb apart showed 54%–58% identity with the Rps1-k-2 protein. These two genes are located in two overlapping *Lotus *BAC clones, LjT02F05 and LjT20J15. Apart from the *Lotus Rps1*-k homolog, no nucleic acid sequences of these two *Lotus *BAC clones showed similarity to sequences of the *Rps1*-k region. In order to identify susceptible haplotype (*rps1*), the *Rps1*-k contig sequence was compared with the available BAC end sequences from the SoyGD database that contains sequences of the cultivar Forrest carrying the susceptible *rps1 *gene [27]. No sequence from LG N of the Forrest haplotype was identified that showed similarity to the *Rps1*-k contig reported here (Figure 3B).

It has been reported that plant disease resistance gene loci exhibit extensive loss of synteny. *R *gene-like sequences frequently lack syntenic map locations between the cereal species rice, barley, and foxtail millet [47]. An effort to clone the rice homolog of the barley *Rpg1 *gene was unsuccessful; because, although the DNA markers flanking *Rpg1 *were syntenic between rice and barley, the region containing the gene is absent in the syntenic rice genome [48]. These observations imply that *R *gene loci evolve faster than the rest of the genomes. This is further supported by comparative sequence analysis conducted in crucifers and grasses [49]. *R *genes may be located in less stable regions of the genome such as telomeric or pericentromeric regions where synteny is poorly conserved [50]. The tomato *Tm-2 *gene resides in a heterochromatic region near the centromere of chromosome 9 [51]. The *Rpg1 *gene is located near the telomere of the short arm of barley chromosome 1 [48]. The tomato *Mi-1 *gene is located at the border region between euchromatin and heterochromatin [52]. The lack of microsynteny of the *Rps1*-k region with the currently available genome sequences and abundance of repeat sequences including retroelements suggested that *Rps1*-k is located in a heterochromatic region which could be pericentromeric.

The *Rps1*-k-1 and *Rps1*-k-2 genes are about 20 kb apart (Figure 3). Most frequently *R *genes are arranged in clusters, and genes within one cluster are mostly derived from a common ancestral gene [4]. This clustering feature is considered to facilitate the expansion of *R *gene numbers and race-specificities through recombination and positive selection [5]. Study of multiple, genetically linked *R *gene families has provided insight into the molecular mechanisms of *R *gene evolution and the generation of novel recognition specificity. Seven family members of *Xa21 *were identified within a 230-kb region [9]. Similarly, seven members of the tomato *I2 *gene family reside in a 90 kb region [53]. The tomato *Cf-2 *locus contains two nearly identical resistance genes in a 17 kb fragment [54]. Both tomato *Cf4 *and *Cf9 *loci comprised of four additional tandemly duplicated paralogous copies within a 36-kb region [55]. Paralogous *R *gene sequences have also been reported to map more distantly. For example, members of the lettuce *Dm3 *family span at least 3.5 Mb with at least 120 kb distance between two gene members [56]. Although in these examples members are evolved from one progenitor gene [4], three distinct CC-NB-LRR gene families were identified in the *Mla *locus within a 240-kb region [7,57]. The potato *R1 *locus also contains three fast evolving CC-NB-LRR genes that undergo frequent sequence exchanges among members of individual groups [58].

Plants have to generate novel resistance specificities to combat the quickly evolved pathogens. This clustering feature can facilitate the expansion of *R *gene numbers and the generation of new *R *gene specificities through recombination and positive selection [5]. Paralogous *R *gene sequences were most likely evolved through unequal recombination. The maize *Rp1 *locus, carrying nine paralogues, is probably the best example of unequal recombination for evolution of tandem paralogous *R *gene sequences [59]. An unequal crossing over between Arabidopsis *RPP8 *and its paralog, *RPHA8 *most likely resulted in *rpp8 *[60]. An unequal crossing over event was detected at the *Rps1*-k region leading to tandem duplication [32].

## Conclusion

Genomes of higher plants vary significantly in their size and complexity because of the existence of a large amount of repetitive sequences. It was observed that the *Rps1*-k region is composed of mostly tandem repeat sequences and retroelements. Several disease resistance genes have been found in the less stable regions of the genome such as telomeric or pericentromeric regions where synteny is poorly conserved. The lack of microsynteny of the *Rps1*-k region with the currently available genome sequences and abundance of repeat sequences in the locus suggest that *Rps1*-k is located in a heterochromatic region that could be pericentromeric.

## Methods

### BAC DNA sequencing

The details of sequencing strategies of the three BACs, GS_18J19, GS_43D16 and GS_99I16 were described previously [1]. The sequence reads generated were assembled using the Phred/Phrap/Consed package [61,62].

### Directional sequencing of GS_43D16

The EZ::TN <*Not*I/KAN-3> transposon insertion BAC clones were generated using the EZ::TN in-Frame Linker insertion kit (Epicentre, Madison, WI). The transposon insertion sites were mapped by *Not*I digestion. Southern hybridization was carried out to physically map the position of transposon insertion in each clone. Both ends of GS_43D16 were used as probes. The 5'-end sequence of GS_43D16 was amplified with primers: (i) GS_43D16 end1F: CTGTAAATTATAAACACATGCCAT and (ii) GS_43D16-end1R: GCTGAATTTCAGTGTAGTGGCGTTTAC. The 3'-end sequence of GS_43D16 was amplified with primers: (i) GS_43D16 end2F: CCCATCCTCATTAATACTTCACACCAC and (ii) GS_43D16 end2R: GTAGTGGAAGTCTATAGTTGTATACCTCTC. BAC DNA was prepared using the alkaline lysis minipreparation procedure. The clones were sequenced in a 96-well plate using either *Not*I/KAN-3 FP-2 or *Not*I/KAN-3 RP-2 primer provided in the EZ::TN in-Frame Linker insertion kit (Epicentre, Madison, WI). Sequencing was conducted at the Iowa State University DNA Facility.

### Gene prediction and sequence analysis

Two gene prediction software packages were used in analyzing the BAC sequences: GENSCAN and GeneMark.hmm ES-3.0 (E – eukaryotic; S – self-training; 3.0 – the version) [34]. The Arabidopsis-based scoring matrix was applied when using GENSCAN. Arabidopsis, maize, rice and *Medicago *were used as model species when GeneMark.hmm was applied. To more accurately predict gene content in the *Rps1*-k region, the predicted genes were further analyzed using different BLAST programs of the NCBI Basic Local Alignment Search Tool (Blast) server [63]: (i) discontiguous Mega Blast program with entrez query limited to Arabidopsis, lotus, *Medicago *and soybean; (ii) Blastn against the soybean EST database; (iii) BlastX and (iv) BlastP. Soybean EST distribution on the BAC sequence was evaluated using the BlastN program. The simple repeat sequences and tandem repeat sequences were identified using Sputnik and tandem repeats finder program [64], respectively.

## Authors' contributions

HG carried out all the studies presented in the paper. MKB conceived the study and participated in the experiment design and coordination, and helped with drafting and finalizing the manuscript.
